# Identification and validation of 174 COVID-19 vaccine candidate epitopes reveals low performance of common epitope prediction tools

**DOI:** 10.1038/s41598-020-77466-4

**Published:** 2020-11-24

**Authors:** Marek Prachar, Sune Justesen, Daniel Bisgaard Steen-Jensen, Stephan Thorgrimsen, Erik Jurgons, Ole Winther, Frederik Otzen Bagger

**Affiliations:** 1 Center for Genomic Medicine, Rigshospitalet, Copenhagen University Hospital, Copenhagen, Denmark; 2 Bioinformatics Centre, Department of Biology, University of Copenhagen, Copenhagen, Denmark; 3 Immunitrack ApS, Copenhagen, Denmark; 4 INTAVIS Peptide Services GmbH & Co.KG, Waldhäuser Str. 64, 72076 Tübingen, Germany; 5 Department of Applied Mathematics and Computer Science, Technical University of Denmark, 2800 Kgs. Lyngby, Denmark; 6 Department of Biomedicine, UKBB Universitats-Kinderspital, Basel, 4031 Basel, Switzerland; 7 Swiss Institute of Bioinformatics, Basel, 4053 Basel, Switzerland

**Keywords:** Antigen processing and presentation, MHC, Machine learning, Viral infection, Software, Proteome informatics

## Abstract

The outbreak of SARS-CoV-2 (2019-nCoV) virus has highlighted the need for fast and efficacious vaccine development. Stimulation of a proper immune response that leads to protection is highly dependent on presentation of epitopes to circulating T-cells via the HLA complex. SARS-CoV-2 is a large RNA virus and testing of all of its overlapping peptides in vitro to deconvolute an immune response is not feasible. Therefore HLA-binding prediction tools are often used to narrow down the number of peptides to test. We tested NetMHC suite tools' predictions by using an in vitro peptide-MHC stability assay. We assessed 777 peptides that were predicted to be good binders across 11 MHC alleles in a complex-stability assay and tested a selection of 19 epitope-HLA-binding prediction tools against the assay. In this investigation of potential SARS-CoV-2 epitopes we found that current prediction tools vary in performance when assessing binding stability, and they are highly dependent on the MHC allele in question. Designing a COVID-19 vaccine where only a few epitope targets are included is therefore a very challenging task. Here, we present 174 SARS-CoV-2 epitopes with high prediction binding scores, validated to bind stably to 11 HLA alleles. Our findings may contribute to the design of an efficacious vaccine against COVID-19.

## Introduction

2019-nCoV (SARS-CoV-2) was first reported in Wuhan, China, on 31 December 2019, following a series of unexplained pneumonia cases^1^. Currently, the disease is rated as a global pandemic by The World Health Organization with case reports from all continents, as of 4 October 2020 the disease has infected more than 34 million people and has claimed more 1 million lives globally^2^. Vaccine development is of high priority, and a number of public and private initiatives are focused on this task^3^. Many of the ongoing vaccine development efforts are focused on raising an immune response against the spike protein. However, the spike protein only makes up 1/8 of the SARS-CoV-2 genome, so this vaccine strategy may inadvertently miss a lot of potential immune reactivity. SARS-CoV-2 has a large proteome^4^. Immune deconvolution to identify T cell epitopes will require initial filtering to assess which SARS-CoV-2-derived peptides are likely to bind a given HLA allele and to be presented on the surface of infected cells from where it can activate passing T cells. The core binding groove of most MHC class I molecules can accommodate 9 amino acid residues, with some variation or suspected impact of flanking positions^5,6^. MHC class II has been described to bind longer peptides (up to 13–25 residues long) interacting with the open binding groove^7^. Providing the possibility for further inspection of the importance of the binding motif and its flanking regions.

Several computational tools (a selection is presented in Table 1) have been developed that can predict the binding of peptides to HLA. Traditionally, these tools were trained using data from affinity assays^8^, but more recently many of them also incorporate data from peptides identified by HLA ligandome analysis. Most tools rely on small neural networks (NN) or variations of position-specific weight matrices (PSSM), to calculate the probability of a peptide matching a consensus motif or model.

**Table 1:** Current best-performing or novel HLA prediction tools^10^.

HLA	Tool	Alleles available*	Year	Algorithm	Web server	References
Class I	NetMHC 4.0	9/10	2003	NN	Yes	^18^
	IEDB-AR Consensus	9/10	2006	Cons	Yes	^19^
	ConvMHC	6/10	2017	NN	Yes	^20^
	DeepHLAPan	10/10	2019	NN	Yes	^21^
	HLAthena	10/10	2020	NN	Yes	^22^
	MixMHCpred 2.0.2	10/10	2017	PSSM	No	^23^
	MHCFlurry 1.3.0	8/10	2018	NN	No	^24^
	NetMHCcons 1.1	10/10	2012	Cons	Yes	^25^
	NetMHCpan_BA 4.0	10/10	2017	NN	Yes	^14^
	NetMHCpan_EL 4.0	10/10	2017	NN	Yes	^14^
	NetMHCstab 1.0	10/10	2014	NN	Yes	^26^
	PickPocket 1.1	10/10	2009	PSSM	Yes	^27^
	PSSMHCpan 1.0	10/10	2017	PSSM	No	^28^
	SMM 1.0	8/10	2005	PSSM	Yes	^29^
	SMMPMBEC 1.0	8/10	2009	PSSM	Yes	^30^
Class II	IEDB-AR Consensus	1/1	2008	Cons	Yes	^31^
	NetMHCIIpan 3.2	1/1	2018	NN	Yes	^32^
	NN_Align 2.3	1/1	2018	NN	Yes	^32^
	SMM-align 1.1	1/1	2007	PSSM	Yes	^33^
	Sturniolo 1.0	1/1	1999	PSSM	Yes	^34^

Webservers checked on 2 March 2020, NN: Neural network, Cons: Consensus, PSSM: Position specific scoring matrix.

*Availability as a fraction of alleles included in study (10 HLA class I and 1 of HLA class II).

NetMHC tools (such as NetMHC, NetMHCII, NetMHCpan, NetMHCIIpan and others) have been under constant development and have consistently performed well throughout the last decade^9–12^. Several tools are restricted in terms of which alleles are available for prediction, in particular for MHC class II. This restriction is primarily determined by the availability of training data, for which the largest public collection is currently the Immune Epitope Database (IEDB)^13^. Attempts to overcome this limitation have been made via sequence-to-sequence predictions, most notably for NetMHCpan^14^. A number of recent publications makes use of prediction tools to suggest vaccine candidate epitopes for SARS-CoV-2^15–17^.

To assess whether current peptide-HLA prediction tools could be suitable for identification of epitopes relevant in a vaccine against SARS-CoV-2, we tested binders predicted by the netMHC tools, using a new peptide-MHC complex-stability assay NeoScreen on ten HLA class I alleles and one HLA class II allele. The selection of class I alleles broadly covers populations across different ethnic origins (Table S1). Subsequently, we chose all tools included in the benchmark recently reviewed by Mei *et al.*^10^, excluding the three tools with the lowest performance (MHCnuggets, HLA-CNN and RANKPEP), as well as SYFPEITHI which could not be brought to run on our system. Furthermore, we added newly developed tools such as HLAthena and DeepHLAPan and a standard tool SMM 1.0 to offer a comprehensive representation of the current prediction tool landscape (Table 1). Most of the selected tools are periodically tested in the IEDB Automated Benchmark^35,36^.

We found that algorithmically predicting binding between epitopes from SARS-CoV-2 and HLA outputs many complexes that turned out to exhibit low stability. Such peptides are thus very unlikely to elicit an immune response against SARS-CoV-2 and are therefore unsuitable for vaccine development. To investigate if this finding was a result of the quality of available training data, we constructed a proof-of-concept prediction model for HLA-A*02:01, which we trained on 2193 historic in-house stability data points, and found that it outperforms other tools. Training data was primarily human cancer-derived or based on random sequences. SARS-CoV-2 peptides that we validated as binding or non-binding in this study are freely available for use to assist in vaccine design against COVID-19.

## Results

We set out to identify peptides with epitope potential in a future COVID-19 vaccine. We commenced by translating the reference sequence of SARS-CoV-2 (ACCESSION MN908947, VERSION MN908947.3) to a protein-coding sequence. Then we predicted potential epitopes in a sliding window of 9 for HLA class I and of 12 for class II using netMHC tools (netMHC/II and “-pan” versions, when allele was not available), for details see Data S1. We identified the top 94 predicted peptides for 11 HLA alleles (94 × 11 = 1034) and went further to validate the binding of these 94 peptides to each allele in an in vitro MHC-peptide complex stability assay (NeoScreen). We removed eight peptides that were synthetically introduced when translating the DNA sequence to protein sequence. Of the remaining 1026 peptides we observed a high degree of overlap between different alleles, resulting in 777 unique peptides. In order to first assess potential variability across the stability measurements we made replicate measurements (n = 4) of 30 randomly selected peptides over 8 different HLA alleles. Each peptide was measured with urea in 4 different concentrations (0 M, 2 M, 4 M, 6 M), and we observed an average standard deviation between replicates of 0.10 with an average mean of 0.56 (Figure S1). All remaining experiments were performed in duplicate for all concentrations. We found that 174 of the 777 unique peptides formed a stable peptide-HLA complex. Of these 174 peptides, currently 98 were previously measured and deposited in IEDB either as a 9-mers or as a substring of a longer peptide, 3 peptides were reported in recent studies^37–39^ but not deposited in the IEDB and 73 remaining peptides are novel. The overlap with peptides deposited in IEDB clearly points out to cross-reactivity between SARS-CoV and SARS-CoV-2, this cross-reactivity has been described in a recent study showing that individuals infected with SARS retained long-lasting memory T cells reacting to the N protein of SARS-CoV, as well as N protein of SARS-CoV-2^40^. Since the completion of our measurements and data search there has been rapid development and many new studies have emerged. The full list of predicted binders (excluding synthetic peptides) can be found in the Supplementary materials (Supplementary Data S1).

To further address whether alternative prediction tools would have higher concordance with measured stability, we performed predictions for all tools listed in Table 1. Predictions for the 19 different tools were performed either through their web server or a stand-alone version, (see Materials and methods section for details). Furthermore, using in-house stability data, we developed PrdX 1.0, a prediction tool for a single allele HLA-A*02:01, where all other tools performed poorly.

We assessed the false positive rate for each tool via Receiver Operating Characteristic (ROC) curves, and their Area under curve (AUC) for all alleles that had more than 10 binders.

**Figure 1:**
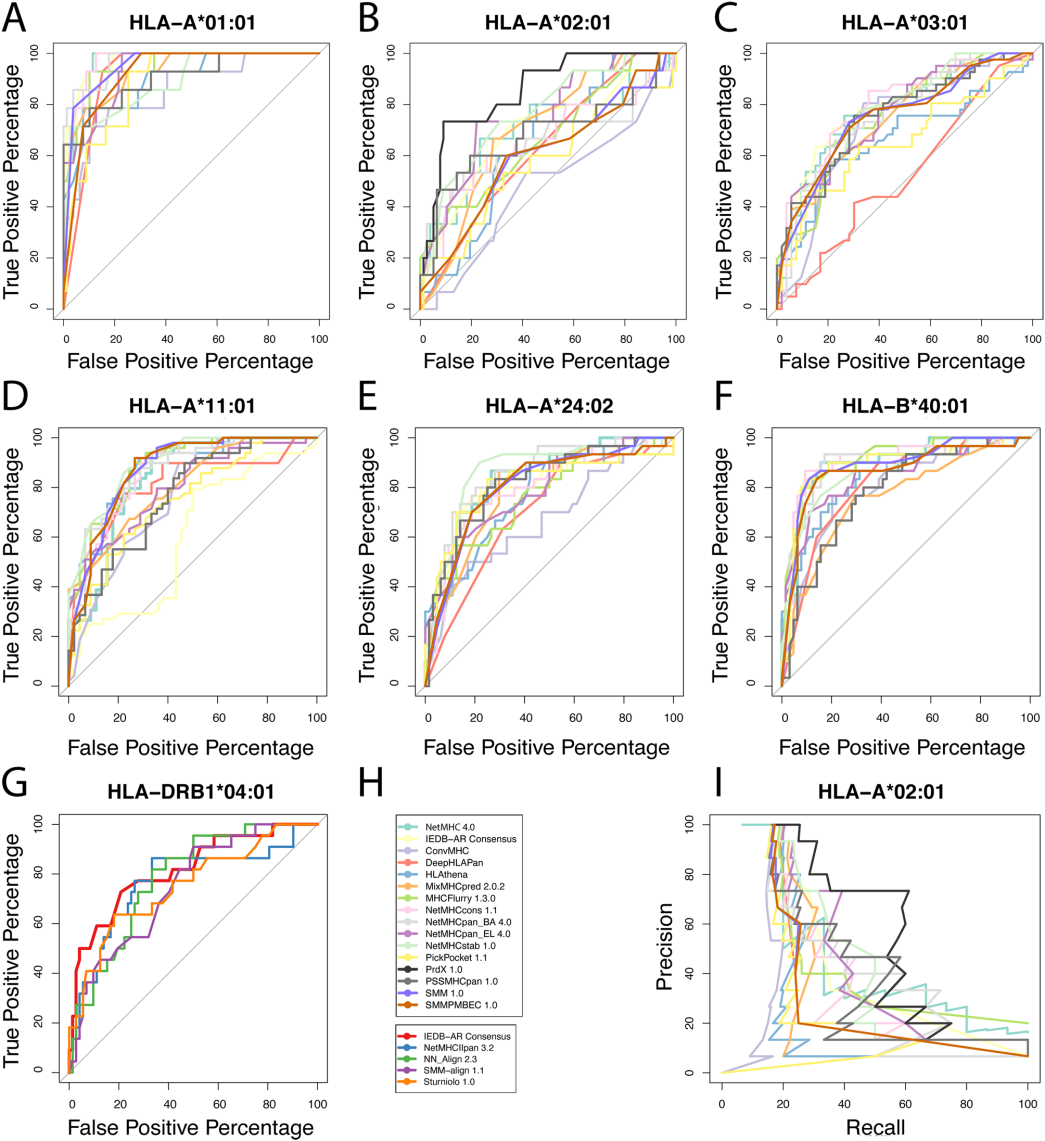
ROC curves for each allele that bound more than 10 peptides stably (subplots **A**, **B**, **C**, **D**, **E**, **F**, **G**), (**H**) tools used in the benchmark, upper box—HLA class I, lower box—HLA class II (IEDB-AR Consensus is available for both), (**I**) precision-recall curves for HLA-A*02:01. Corresponding area under curve (AUC) values are listed in Table 2.

The analysis revealed that NetMHC 4.0 achieved the highest score for allele HLA-A*01:01 (AUC = 97.47; Fig. 1A), closely followed by NetMHCcons 1.1, NetMHCpan_BA 4.0 and IEDB-AR Consensus. PrdX 1.0 scored highest for HLA-A*02:01 (AUC = 85.54; Fig. 1B), NetMHCcons 1.1 scored highest for HLA-A*03:01 (AUC = 79.25; Fig. 1C), and MHCflurry 1.3.0 performed best for HLA-B*40:01 (AUC = 91.06; Fig. 1F). NetMHCstab 1.0 was the only tool that achieved the highest score for more than 1 allele: HLA-A*11:01 and HLA-A*24:02 (AUC = 89.80; 86.03; Fig. 1D,E, respectively). Out of the tools tested for HLA class II, IEDB-AR Consensus achieved the highest score for HLA-DRB1*04:01 (AUC = 81.31; Fig. 1G). Table 2 provides all AUC values, and the best result obtained for each allele is marked in bold. Notably, in the case of HLA-A*02:01 we observed particularly poor performance among all tested tools despite the extensive amount of data available for this allele.

**Table 2:** AUC values for ROC curves from Fig. 1 for alleles with more than 10 stable complexes.

Tool/allele	A*01:01	A*02:01	A*03:01	A*11:01	A*24:02	B*40:01	DRB1*04:01
NetMHC 4.0	**97.47**	70.3	77.06	83.81	82.96	89.76	–
IEDB-AR Consensus	97.06	69.44	77.36	87.05	83.89	90.03	**81.31**
ConvMHC	85.13	47.71	72.53	76.33	66.88	79.80	–
DeepHLAPan	91.82	62.90	52.49	80.84	69.87	80.95	–
HLAthena	89.74	65.41	66.54	87.41	76.48	83.08	–
MixMHCpred 2.0.2	92.54	70.04	75.29	80.82	78.68	76.29	–
MHCFlurry 1.3.0	94.48	66.88	75.52	88.46	76.24	**91.06**	–
NetMHCcons 1.1	97.42	65.93	**79.25**	86.21	79.52	90.25	–
NetMHCpan_BA 4.0	97.15	65.84	76.32	86.76	85.40	88.79	–
NetMHCpan_EL 4.0	93.40	75.89	75.84	80.11	78.36	84.27	–
NetMHCstab 1.0	89.15	76.75	77.98	**89.80**	**86.03**	86.13	–
PickPocket 1.1	88.65	57.32	65.53	75.03	80.93	88.44	–
PrdX 1.0	–	**85.54**	–	–	–	–	–
PSSMHCpan 1.0	90.33	67.97	75.75	76.62	82.12	77.94	–
SMM 1.0	95.25	60.09	75.15	87.26	80.26	88.82	–
SMMPMBEC 1.0	92.13	60.48	75.36	87.26	80.13	86.34	–
NetMHCIIpan 3.2	–	–	–	–	–	–	76.63
NN_Align 2.3	–	–	–	–	–	–	78.14
SMM-align 1.1	–	–	–	–	–	–	74.42
Sturniolo 1.0	–	–	–	–	–	–	75.19

First 6 six columns contain HLA class I, last column contains HLA class II. Only four tools were tested for HLA class II. PrdX 1.0 is only available for allele A*02:01. Highest value for each allele is marked in bold.

**Figure 2:**
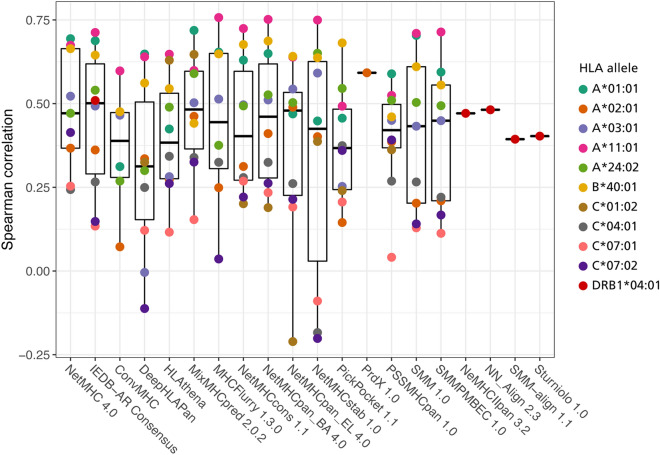
Plot of Spearman correlation coefficient between predicted values and results of NeoScreen stability assay for each available allele. Each colour represents an individual allele. Boxplot whiskers accord for 1.5 distance between the median and quartile hinges.

To assess the correlation between the predicted and measured peptide-HLA complexes, Spearman correlation coefficient (SCC) was calculated for all alleles. This revealed significant inconsistencies in performance depending on the predicted allele. PSSMHCpan 1.0 displayed the highest consistency, taking into account its coverage (Table 1), but the correlation median scored lower than other tools such as IEDB-AR Consensus, MixMHCpred 2.0.2, NetMHCpan_EL 4.0 or PrdX 1.0. The results of the Spearman correlations are summarized in Fig. 2.

Lastly to compare the performance of our benchmark we calculated the average percentile score as described at the IEDB website^35^ for all tools and alleles where we had both AUC and SCC available. Comparison between overlapping tools in IEDB Automated Benchmark and our study can be found in Supplementary materials (Table S2).

## Discussion

Here we benchmark a number of tools to identify epitopes for SARS-CoV-2 virus and validate via stability assay the binding of candidate epitopes to 10 alleles of HLA class I and one allele of HLA class II. We find that the false positive rate is high for all tested tools when testing binding stability for predicted HLA-binding peptides from SARS-CoV-2 virus. This creates a challenge for vaccine development efforts, especially for the design of epitope vaccines, where only a limited number of epitopes may be included. Furthermore, it highlights the risk for failed vaccine design (for any pathogen or disease) if predicted HLA-binding protein regions in reality do not bind stably and allow immune presentation and response.

We observed, remarkably, that all tools tested performed poorly for HLA-A*02:01, which is the allele with most training data available^41^. Based on our observations we hypothesise that publicly available training data is not of high enough quality. This is supported by the fact that AUC and Spearman correlations indicate that performance seems to correlate with the alleles and not the tools; thus, suggesting that either the training data or the difficulty of modelling the allele is responsible for poor predictions. To test the hypothesis that training data is limiting for tool performance, we trained a vanilla NN on only 2193 historic in-house stability measurements and found that our model outperformed all tested prediction tools in this setting. This observation could also be explained by more similar data distributions between test and training data for PrdX 1.0.

We identified 174 potential SARS-CoV-2 vaccine candidate peptides, out of which 98 have been previously deposited in IEDB following various studies^13,42–46^. The majority of the previously deposited peptides were measured in one or multiple affinity assays and reached low Kd (< 50 nM) values, indicating strong affinity. Additionally, 9 of these peptides were previously measured in another stability assay and were recognised as stable binders^47^, independently confirming our approach and measurements. Recently, new T cell studies uncovered a large overlap with stable peptides from our assay: 60 peptides showed a positive T cell response in one or more performed studies^37–39^. Such a result not only reveals the true potential of complex-stability assays but also contributes to collective findings about cross-reactivity of SARS-CoV and SARS-CoV-2.

In conclusion, we make freely available 174 COVID-19 epitopes that we have predicted and validated in vitro to be HLA-binding. We hope that this contribution will aid the development of a vaccine against SARS-CoV-2. We performed a benchmark analysis of 19 tools on 777 peptides that were predicted by state-of-the-art prediction tools from netMHC to be binders and revealed high false positive rates for all benchmarked tools. We observed improved performance after training our own prediction tool PrdX 1.0 on allele HLA-A*02:01 using in-house generated stability data. Our findings suggest that the performance of current state-of-the-art epitope prediction tools are impacted by the varying quality of publicly available data.

## Materials and methods

Nineteen prediction tools tested on a relevant dataset of peptides from the SARS-CoV-2 genome (assembly MN908947.3). The genome sequence was downloaded from the NCBI database (https://www.ncbi.nlm.nih.gov/nuccore/MN908947.3)^4^. Using NetMHC tools we predicted the top 94 peptides for HLA-A*01:01, HLA-A*02:01, HLA-A*03:01, HLA-A*24:02, HLA-B*40:01, HLA-C*04:01, HLA-C*07:01, HLA-C*07:02 (netMHC 4.0), HLA-C*01:02 (NetMHCpan 4.0) and HLA-DRB1*04:01 (NetMHCII 2.3). Subsequently, the peptides were analysed for binding stability to the respective HLA allele. Taking into account the cross-reactivity between the two alleles, peptides predicted to bind HLA-A*03:01 were also measured on HLA-A*11:01. For HLA-DRB1*04:01 we increased the synthesized peptides from length 9 to 12 in order to account for the effect of flanking regions to the core binding sequence.

Peptides were synthesised using standard Fmoc solid-phase synthesis on a modified cellulose support as solid support according to the SPOT synthesis protocol, starting with the acid labile Ramage linker.

After synthesis, peptides were cleaved off the membranes using 95% trifluoroacetic acid (TFA), 3% triisopropylsilane (TIS) & 2% H_2_O. Peptides were then precipitated with diethylether and washed with methyl-tert-butylether.

Peptides were subsequently dissolved in a proprietary mixture and dried under vacuum using a speed vac. Finally, 5% of all peptides were analysed by MALDI-TOF to confirm correct molecular weight. The anticipated yield per spot was 50 μg.

### NeoScreen assay

The NeoScreen stability assay utilises urea denaturation to assess peptide-MHC complex stability. Briefly, peptides were dissolved in 200 µl DMSO with 1 mM β-mercaptoethanol and subsequently diluted into an assay buffer in 96 well plates at a final concentration of 2 µM. Positions A1 and H12 were reserved for a mixture of reference peptides with known stable binding to the MHC of interest. MHC I was diluted into an assay buffer with beta 2 microglobulin (b2m) and added at a 1:1 ratio to diluted peptides. For MHC II, the urea-denatured alpha and beta chains were diluted into an assay buffer and added at a 1:1 ratio to diluted peptides. The concentration of MHC depended on the actual chain, but final concentrations were in the range of 2–10 nM (hence peptide was added in excess). Upon folding, peptide-MHC complexes were transferred to 384 well plates where they were challenged with 4 different urea concentrations. Following the period of urea-induced stress the plates were developed in a conventional ELISA as described previously^48,49^. The ABS450 nm signals from the 4 different wells were averaged and normalised to the included reference to the included reference peptides in wells A1 and H12.

Unlike other previously developed assays NeoScreen offers a high-throughput process without a need to use iodine labelled b2m or FACS based quantification^50,51^. When compared with a recently developed method which uses thermal denaturation and differential scanning fluorimetry a same stability trend was found, where MART-1 wt had lowest stability, Tyrosinase and HTLV-TAX (NeoScreen reference peptide for HLA-A*02:01) had very high stability^51^.

### Benchmarking of tools

Table 1 provides a summary of tools tested in this benchmark analysis. It features the year of their development, the algorithm used, web server availability and a reference. Most of the tested tools are available at the IEDB Analysis Resource web page (https://tools.iedb.org/main/) and were run through their web interface (https://tools.iedb.org/mhci/ or https://tools.iedb.org/mhcii/). MixMHCpred 2.0.2, MHCflurry 1.3.0 and PSSMHCpan 1.0 were downloaded from their respective GitHub pages (https://github.com/GfellerLab/MixMHCpred, https://github.com/openvax/mhcflurry, https://github.com/BGI2016/PSSMHCpan, respectively). ConvMHC, DeepHLApan and HLAthena were used from their privately hosted web servers (https://jumong.kaist.ac.kr:8080/convmhc, https://biopharm.zju.edu.cn/deephlapan/, https://hlathena.tools/, respectively).

All tested peptides were subjected to in silico predictions (with each prediction tool) regarding their available allele. Predictions were compared against measured stability determinations obtained through the NeoScreen assay. Measurements were normalised to an allele-specific reference peptide (stability = 100). The list of reference peptides used is available in Supplementary materials (Table S3). The threshold for a stable binder was set to 60. Predictions were subsequently evaluated according to commonly used metrics such as the Receiver Operating Characteristic (ROC) and its Area Under Curve (AUC) to visualise the relationship between sensitivity and specificity, corresponding equations can be found in the Supplementary methods. Spearman correlation was also used to compare the ranked correlation of predicted and measured data.

### PrdX

To assess the performance of predictors trained on stability data we used PyTorch^52^ to train a fully connected, feed-forward neural network with 64 and 32 hidden units on historic in-house stability data from allele HLA-A*02:01. This data contains a mixture of human cancer-related stability measurements and measurements made on synthetic random peptides. We used BLOSUM62 matrix for encoding, simple network architecture, train-test split and early stopping for training.

## Supplementary information

Supplementary Information.

## Data Availability

All epitopes are available at the vendor webpage (www.immunitrack.com) and in Supplementary materials (Data S2).
