# Supplementary material for: Identification and validation of 174 COVID-19 vaccine candidate epitopes reveals low performance of common epitope prediction tools
Source: Sci Rep. 2020 Nov 24;10:20465. doi: 10.1038/s41598-020-77466-4 (PMC7686376; doi:10.1038/s41598-020-77466-4)
Supplement: Supplementary file 1 — Supplementary Information. [file 41598_2020_77466_MOESM1_ESM.pdf]

Supplementary Materials for

# Identification and validation of 174 COVID-19 vaccine candidate epitopes reveals low performance of common epitope prediction tools

Marek Prachar[1,2,3], Sune Justesen[3], Daniel Bisgaard Steen-Jensen[3], Stephan Thorgrimsen[3], Erik Jurgons[4], Ole Winther[1,2,5], and Frederik Otzen Bagger[1,6,7,*]

1. Center for Genomic Medicine, Rigshospitalet, Copenhagen University Hospital, Copenhagen, Denmark
2. Bioinformatics Centre, Department of Biology, University of Copenhagen, Copenhagen, Denmark
3. Immunitrack ApS, Copenhagen, Denmark
4. INTAVIS Peptide Services GmbH & Co.KG, Waldhäuser Str. 64, 72076 Tübingen, Germany
5. Department of Applied Mathematics and Computer Science, Technical University of Denmark, 2800 Kgs. Lyngby, Denmark
6. UKBB Universitats-Kinderspital, Department of Biomedicine, Basel, 4031 Basel, Switzerland
7. Swiss Institute of Bioinformatics, Basel, 4053 Basel, Switzerland

* Correspondence to Frederik Otzen Bagger, frederik.otzen.bagger@regionh.dk

This PDF file includes:

Supplementary methods
Figure S1
Table S1
Table S2
Table S3
Captions for Data S1-S2

Supplementary methods

Analysis of predictions

To produce the ROC graphs and to calculate their AUC we used RStudio Version 1.2.5033, R version 3.6.2, pROC 1.16.1 package. The same software was used to calculate and plot Spearman correlations, using the ggplot2 3.2.1 package.

PrdX

The software was written in Python Version 3.7.3, NN architecture was built using PyTorch Version 1.3.0. For training we used a learning rate of 0.003 and used the Adam optimizer.

ROC metrics

$$Sn = \frac{TP}{TP + FN} = 1 - \frac{FN}{P} \qquad 0 \leq Sn \leq 1$$

$$Sp = \frac{TN}{TN + FP} = 1 - \frac{FP}{N} \qquad 0 \leq Sp \leq 1$$

$$FPR = \frac{FP}{FP + TN} = 1 - Sp \qquad 0 \leq FPR \leq 1$$

$$Pr = \frac{TP}{TP + FP} \qquad 0 \leq Pr \leq 1$$

where Sn, Sp, FNR and Pr stands for sensitivity (recall), specificity, false negative rate and precision, respectively. P, FN, N and FP represent the numbers of positives, false negatives, negatives and false positives, respectively. Rates are converted into percentages in the plots in the manuscript.

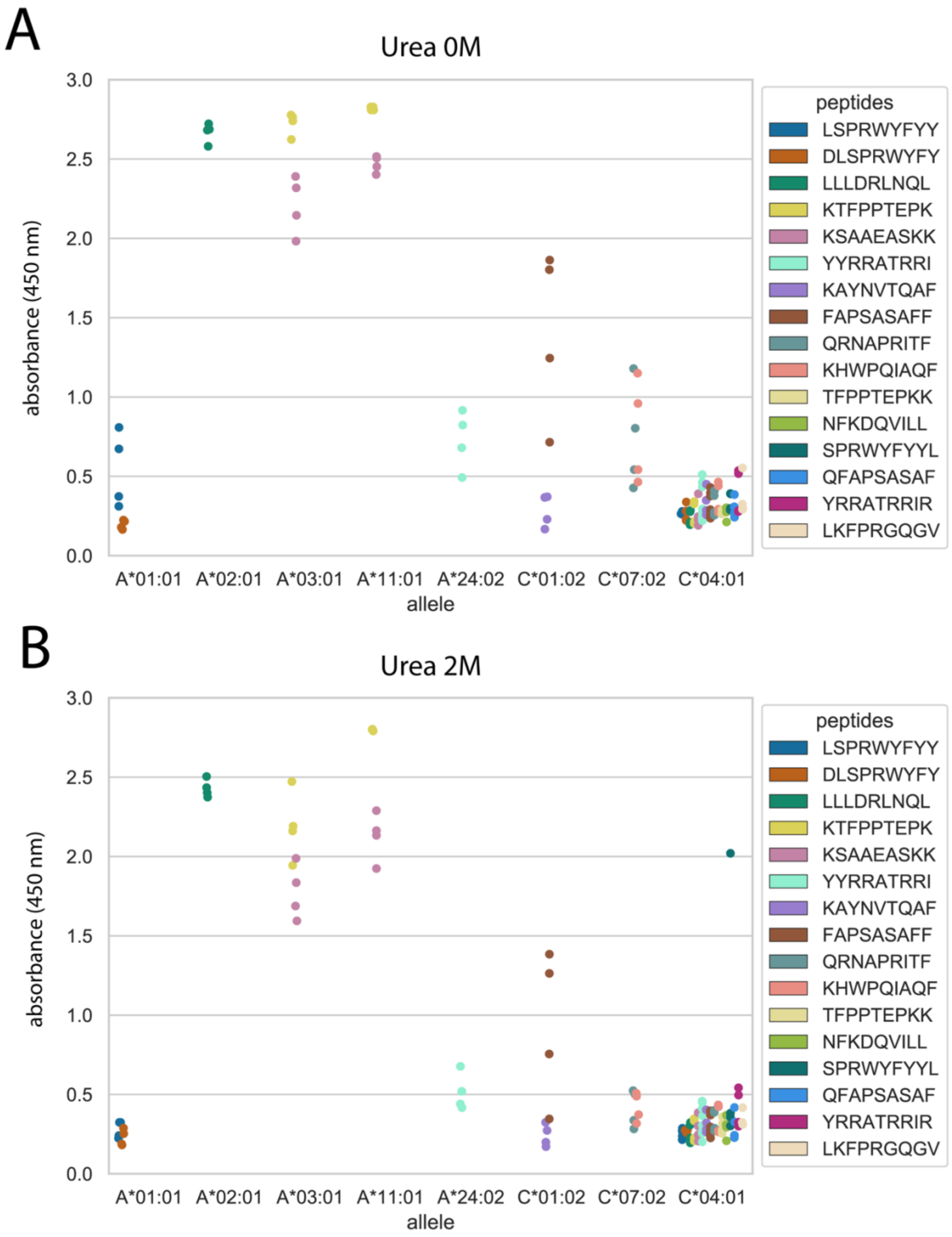

A
Urea 0M
absorbance (450 nm)
3.0
2.5
2.0
1.5
1.0
0.5
0.0
A*01:01
A*02:01
A*03:01
A*11:01
A*24:02
C*01:02
C*07:02
C*04:01
allele
peptides
LSPRWYFYY
DLSPRWYFY
LLLDRLNQL
KTFPPTEPK
KSAAEASKK
YYRRATRRI
KAYNVTQAF
FAPSASAFF
QRNAPRITF
KHWPQIAQF
TFPPTEPKK
NFKDQVILL
SPRWYFYYL
QFAPSASAF
YRRATRRIR
LKFPRGQGV
B
Urea 2M

C

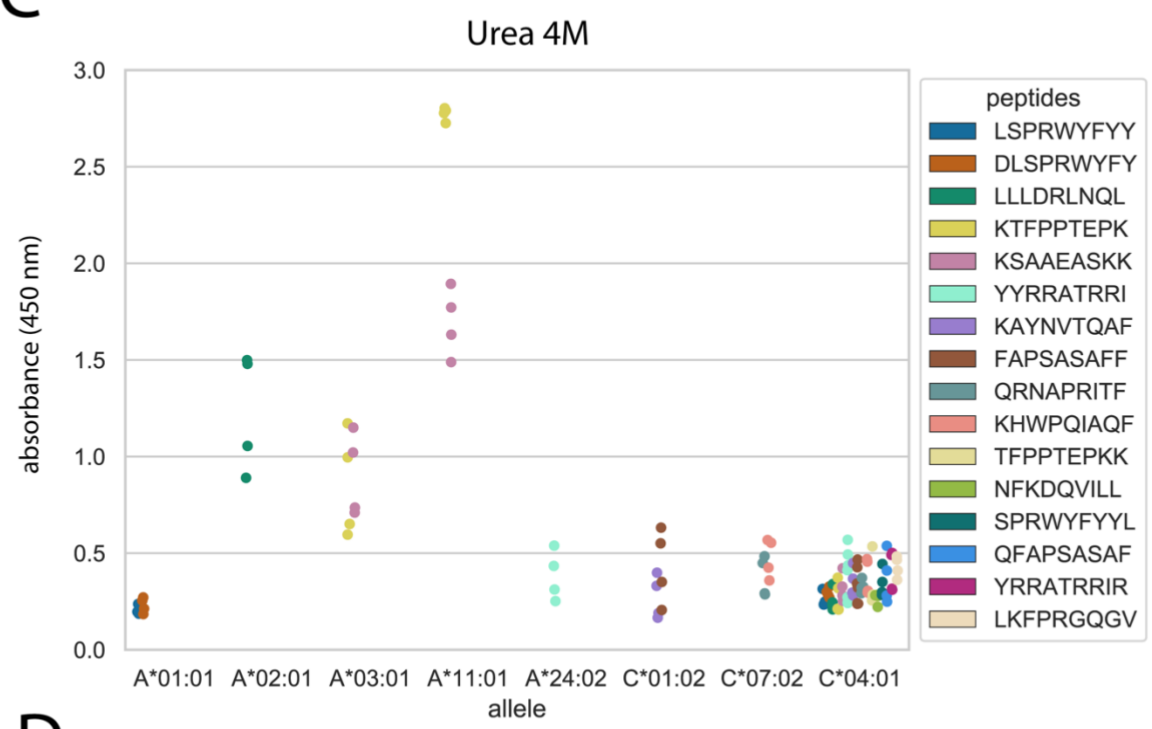


D

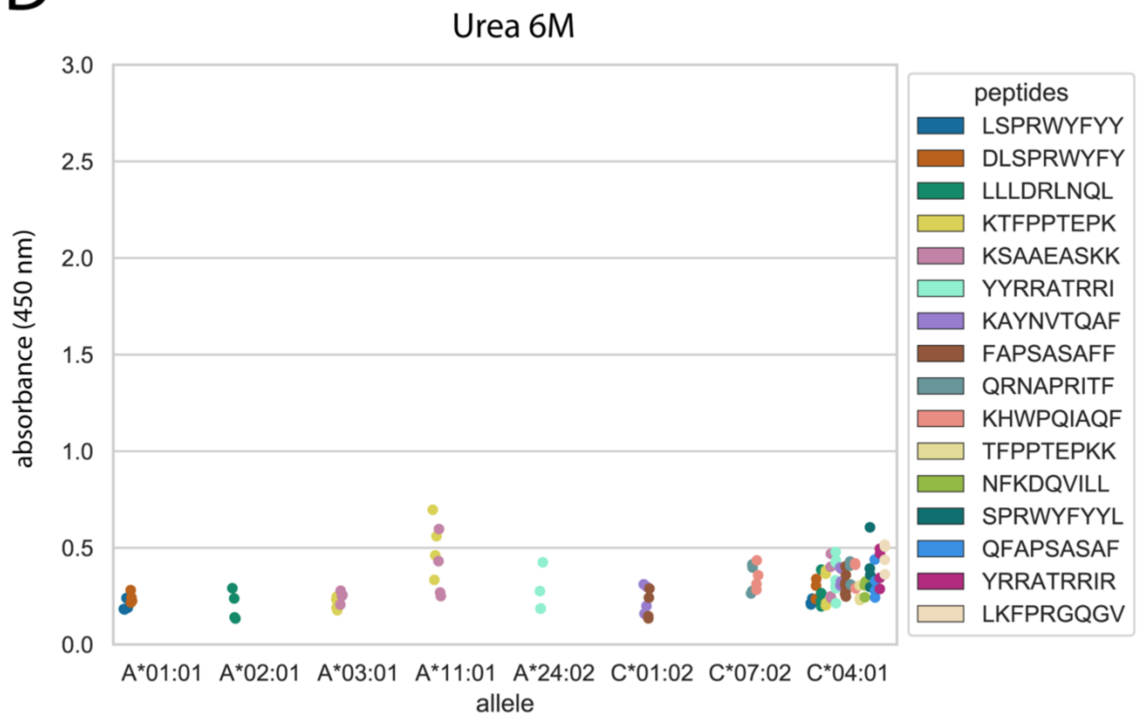


Figure S1. NeoScreen stability assay replicate analysis (n=4), each dot represents a measurement, colored by peptide. Some peptides were measured for multiple alleles. A), B), C), D) correspond to different urea molarity - 0, 2, 4, 6, respectively. Most measurements fall close together, with an average standard deviation of 0.10 and with an average mean of 0.56 between replicates. Majority of the experiments were performed on allele HLA-C*04:01.

Table S1. The prevalence across four different ethnic groups of the selected HLA class I alleles.[1]

| *Allele* | *Caucasian (%)* | *Black (%)* | *Hispanic (%)* | *Asian or Pacific Islander (%)* |
|---|---|---|---|---|
| *A*01:01* | 27 | 10 | 11 | 5 |
| *A*02:01* | 45 | 22 | 37 | 18 |
| *A*03:01* | 23 | 18 | 14 | 3 |
| *A*11:01* | 15 | 2 | 11 | 38 |
| *A*24:02* | 12 | 2 | 24 | 33 |
| *B*40:01* | 13 | 1 | 2 | 15 |
| *C*01:02* | 5 | 1 | 9 | 27 |
| *C*04:01* | 21 | 29 | 25 | 14 |
| *C*07:01* | 27 | 25 | 20 | 1 |
| *C*07:02* | 21 | 18 | 24 | 33 |

Table S2. Benchmark comparison, AUC and SCC percentile ranks were calculated per benchmark performance of each included tool. Then the average of AUC and SCC was calculated.[2]

| *Tool \ Benchmark* | *IEDB (%)*[2] | *Current study (%)* |
|---|---|---|
| MHCflurry 1.2.0/1.3.0 | 80 | 63 |
| NetMHCpan 4.0 | 70 | 67 |
| NetMHC 4.0 | 70 | 72 |
| NetMHCcons 1.1 | 60 | 67 |
| SMMPMBEC 1.0 | 47 | 46 |
| IEDB-AR Consensus | 39 | 77 |
| PickPocket 1.1 | 38 | 34 |
| SMM 1.0 | 37 | 52 |

Table S3. List of reference peptides used for their corresponding allele.

| HLA Allele | Sequence | Reference |
|---|---|---|
| *A*01:01* | VTEHDTLLY | http://www.iedb.org/epitope/71290 |
| *A*02:01* | VLDFAPPGA | Wilms tumor antigen 1 |
| *A*03:01* | AVAHKVHLMYK | https://www.iedb.org/epitope/419554 |
| *A*11:01* | AVFDRKSDAK | https://www.iedb.org/epitope/5316 |
| *A*24:02* | AYAQKIFKIL | https://www.iedb.org/epitope/5731 |
| *B*40:01* | REDQWCGSL | https://www.iedb.org/epitope/53476 |
| *C*01:02* | QYDPVAALF | http://www.iedb.org/epitope/52886 |
| *C*04:01* | QYDPVAALF | http://www.iedb.org/epitope/52886 |
| *C*07:01* | YLHARLREL | Identified by previous screening |
| *C*07:02* | NYFNRMFHF | Identified by previous screening |
| *DRB1*04:01* | AKFVAAWTLKAAA | https://www.iedb.org/epitope/2192 |

Data S1. COVID-19 INTAVIS-Immunitrack dataset

https://www.immunitrack.com/wp/wp-content/uploads/Covid19-Intavis-Immunitrack-datasetV2.xlsx

Excel file containing results of the NeoScreen assay.

Data S2. Full list of predicted and measured peptides used in the benchmark

https://doi.org/10.5281/zenodo.4277689

Public datasets of all peptides that were predicted by the NetMHC suite tools and subsequently measured. List of peptide sequences for each respective allele. All predictions for each of the benchmarked tools (not all tested tools use the same measure). List of binders (epitopes) with IEDB links and T cell studies overlap.

# References


1. Gonzalez-Galarza, F. F. *et al.* Allele frequency net database (AFND) 2020 update: gold-standard data classification, open access genotype data and new query tools. *Nucleic Acids Res.* (2019) https://doi.org/10.1093/nar/gkz1029.
2. Trolle, T. *et al.* Automated benchmarking of peptide-MHC class I binding predictions. *Bioinformatics* **31**, 2174–2181 (2015).
